# Autophagy-related genes analysis reveals potential biomarkers for prediction of the impaired walking capacity of peripheral arterial disease

**DOI:** 10.1186/s12916-023-02889-5

**Published:** 2023-05-18

**Authors:** Mengjun Dai, Kangbo Li, Mesud Sacirovic, Claudia Zemmrich, Eva Buschmann, Oliver Ritter, Peter Bramlage, Anja Bondke Persson, Ivo Buschmann, Philipp Hillmeister

**Affiliations:** 1 Center for Internal Medicine 1, Department for Angiology, Deutsches Angiologie Zentrum (DAZB), Brandenburg Medical School (MHB) Theodor Fontane, University Clinic Brandenburg, Hochstrasse 29, 14770 Brandenburg an der Havel, Germany; 2 Charité – Universitätsmedizin Berlin, corporate member of Freie Universität Berlin and Humboldt-Universität Zu Berlin, Berlin, Germany; 3 Institute for Pharmacology and Preventive Medicine, Cloppenburg, Germany; 4 Department of Cardiology, University Clinic Graz, Graz, Austria; 5 Department for Cardiology, Center for Internal Medicine I, Brandenburg Medical School Theodor Fontane, University Clinic Brandenburg, Brandenburg an der Havel, Germany; 6 Faculty of Health Sciences, joint Faculty of the Brandenburg University of Technology Cottbus - Senftenberg, the Brandenburg Medical School Theodor Fontane and the University of Potsdam, Brandenburg Medical School Theodor Fontane, Potsdam, Germany

**Keywords:** Autophagy, Biomarkers, Growth-related oncogene, Neutrophil activating protein2, Peripheral arterial disease, Treadmill testing

## Abstract

**Background:**

The role of autophagy and autophagy-related genes in peripheral arterial disease (PAD) remains unknown and may be of diagnostic and prognostic value. The aim of this study is to investigate the relationship between autophagy and PAD, and identify potential diagnostic or prognostic biomarkers for medical practice.

**Methods:**

Differentially expressed autophagy-related genes in PAD were explored from GSE57691 and validated in our WalkByLab registry participants by quantitative real-time polymerase chain reaction (qRT-PCR). The level of autophagy in peripheral blood mononuclear cells (PBMCs) of WalkByLab participants was assessed by analyzing autophagic marker proteins (beclin-1, P62, LC3B). Single sample gene set enrichment analysis (ssGSEA) was used to evaluate the immune microenvironment within the artery wall of PAD patients and healthy persons. Chemokine antibody array and enzyme-linked immunosorbent assay were used to assess the chemokines in participants’ plasma. Treadmill testing with Gardner protocol was used to evaluate participants’ walking capacity. Pain-free walking distance, maximum walking distance, and walking time were recorded. Finally, a nomogram model based on logistic regression was built to predict impaired walking performance.

**Results:**

A total of 20 relevant autophagy-related genes were identified, and these genes were confirmed to be expressed at low levels in our PAD participants. Western blotting demonstrated that the expression of autophagic marker proteins beclin-1 and LC3BII were significantly reduced in PAD patients’ PBMCs. ssGSEA revealed that most of the autophagy-related genes were strongly correlated with immune function, with the largest number of associated genes showing interaction between cytokine-and-cytokine receptors (CCR). In this context, the chemokines growth-related oncogene (GRO) and neutrophil activating protein2 (NAP2) are highly expressed in the plasma of WalkByLab PAD patients and were significantly negatively correlated with the walking distance assessed by Gardner treadmill testing. Finally, the plasma NAP2 level (AUC: 0.743) and derived nomogram model (AUC: 0.860) has a strong predictive potential to identify a poor walking capacity.

**Conclusions:**

Overall, these data highlight both the important role of autophagy and autophagy-related genes in PAD and link them to vascular inflammation (expression of chemokines). In particular, chemokine NAP2 emerged as a novel biomarker that can be used to predict the impaired walking capacity in PAD patients.

**Supplementary Information:**

The online version contains supplementary material available at 10.1186/s12916-023-02889-5.

## Background

Atherosclerosis of the arteries supplying the legs is commonly labeled as lower extremity peripheral arterial disease (PAD). PAD is a common disorder and the third leading cause of atherosclerotic cardiovascular morbidity, following coronary artery disease (CAD) and stroke [1]. It can manifest as intermittent claudication when walking or, in the most severe form, critical limb ischemia. Limb ischemia defined as chronic ischemic rest pain, ulcers, or gangrene of the lower extremity, which is a major cause of limb amputation, and a harbinger of cardiovascular mortality in PAD [1, 2]. Age, tobacco use, diabetes, hypertension, hypercholesterolemia, and a sedentary lifestyle are the major risk factors for PAD [3].

Autophagy is a cellular recycling process in response to various stressors (e.g., oxidative stress, hypoxia, and starvation). It has been implicated in several fundamental biological processes, including aging, immunity, development, tumorigenesis, vascular disease, cell death and differentiation [4, 5]. In the cardiovascular system, autophagy is a key regulator of homeostasis, responding to physiological and pathophysiological stimuli. Although recycling of cellular organelles is generally viewed as a beneficial process, insufficient and excessive levels of autophagy can lead to premature cell death (apoptosis) [6]. To date, there is increasing evidence that basal autophagy plays an essential role in protecting endothelial cells and smooth muscle cells from cell death and the development of vascular disease, particularly heart failure and atherosclerosis [5, 7]. Current research suggest that autophagy is also a fundamental process for cardiovascular homeostasis, health, and aging [8]. To the best of our knowledge, despite the protective role of autophagy in various diseases, its role in the vascular system is poorly understood and is entirely unknown in the context of PAD [9]. Exploring and uncovering potential autophagy-related genes and the autophagy level in PAD as well as a functional linkage with relevant molecular processes of vascular pathophysiology may provide potential biomarkers for assessing and monitoring disease risk and prognosis.

Recently, Biros et al. [10, 11] uploaded a gene expression omnibus (GEO) dataset GSE57691, which revealed the differentially expressed genes in the artery wall tissue from 49 patients with abdominal aortic aneurysm, 9 patients with chronic lower limb ischemia and 10 healthy controls. In the present study, we reevaluated this dataset for potential implications for lower limb ischemia, which served as the basis for an extensive new analysis. First, we explored the differentially expressed autophagy-related genes in PAD patients from the GSE57691 data set and validated target genes expression in peripheral blood samples from our Lauflabor (WalkByLab) registry participants. Then protein–protein interaction (PPI) analyses, Gene Ontology (GO) and Kyoto encyclopedia of genes and genomes (KEGG) enrichment analysis were performed to investigate the biological functions of these autophagy-related genes. Subsequently, western blot was performed to assess the level of autophagy in peripheral blood mononuclear cells (PBMCs) of WalkByLab participants by analyzing the degradation of autophagic marker proteins (beclin-1, P62, and LC3B). Here, a `pro-inflammatory´ phenotype and immune activation is an important feature of PAD [12]. Therefore, we next performed single sample gene set enrichment analysis (ssGSEA) to investigate the immune microenvironment of the artery wall of PAD patients and link molecular inflammatory processes with autophagy-related genes. Finally, we analyzed and validated potential biomarkers for predicting impaired walking capacity as assessed by Gardner treadmill testing used in clinical practice. In particular, the Gardner treadmill testing is regarded as the gold standard for physical performance in the diagnoses of PAD status, but it requires appropriate equipment, time, and a professionally trained team, which is why it is rarely used in clinical practice. In summary, the aim of this study was to (1) gain insight into the relationship between autophagy effector molecules and PAD, (2) link autophagy to inflammatory processes in the development of PAD, and (3) identify novel biomarkers that can reliably predict walking ability and PAD status in clinical practice.

## Methods

### Autophagy-related genes datasets and microarray data

A total of 232 genes were obtained from The Human Autophagy Database (http://www.autophagy.lu/index.html). The mRNA expression profile dataset of GSE57691 was downloaded from GEO (http://www.ncbi.nlm.nih.gov/geo/). GSE57691 is in GPL10558 platform (Illumina HumanHT-12 V4.0 expression beadchip), which included a gene expression analysis of artery wall tissue from 49 patients with abdominal aortic aneurysm, 9 patients with chronic lower limb ischemia and 10 healthy controls.

### Differentially expressed analysis of autophagy-related genes

The normalized expression matrix of microarray data was downloaded from the GSE57691 dataset. Then the probes were annotated with the annotation files from the dataset. The repeatability of data in GSE57691 was verified by principal component analysis (PCA). The “limma” package of R software was used to identify the differentially expressed autophagy-related genes. Genes with an adjusted *P*-value < 0.05 and |log2fold change (FC)|≥ 1 were considered as differentially expressed genes. The heatmap and volcano plot were conducted using “heatmap” and “ggplot2” packages of R software.

### PPI analysis and correlation analysis of the differentially expressed autophagy-related genes

PPI analysis of differentially expressed autophagy-related genes was analyzed using STRING database (https://string-db.org/) and Cytoscape software (version 3.8.2). The correlation analysis of the differentially expressed autophagy-related genes was identified using Spearman correlation in the “corrplot” package of R software.

### GO and KEGG pathway enrichment analysis of autophagy-related genes

GO and KEGG pathway enrichment analysis were conducted in R software using the package “GO plot”. The GO analysis consisted of cellular component (CC), biological process (BP) and molecular function (MF).

### Exploration of immune microenvironment

The single sample gene set enrichment analysis (ssGSEA) was performed to quantify the immune function levels. The normalized enrichment score that calculated from the ssGSEA was used in the ‘GSVA’ and ‘GSEABase’ R package [13, 14]. The annotated gene set file was obtained from the study of Jie-Ying Liang et al. [15]. We finally quantified the enrichment levels of the 16 immune cells and 13 immune-related pathways in each sample to reveal the immune function of PAD, and the results were expressed as immune scores.

The heat map of the involved immune cells and immune-related pathways was made using the "pheatmap" R package. The boxplot showed the level of immune infiltration in PAD group and healthy control group was made using the "ggpubr" and "reshape2" R package. A Spearman’s rank correlation analysis heatmap, which revealed the correlation of different immune cells infiltration levels and immune-related pathways, was performed using the "corrplot" R package. The correlation of autophagy-related genes expression and immune function was analyzed using the "ggcorrplot" R package.

### Participants and their clinical characteristics

A total of 179 participants were selected from the WalkByLab-Registry database. These are the patients who presented to the WalkByLab center Brandenburg/Havel (Brandenburg Clinic, Brandenburg Medical School) Germany between October 2020 and August 2022. The WalkyByLab aims to interdisciplinarily screen, diagnose and follow-up patients with cardiovascular disease (www.lauflab.de). The WalkByLab register trial protocol was reviewed and approved by the ethical committee of the Cottbus Medical Association (Landesärztekammer Cottbus, study number of the ethics committee: AS 74(bB)/2018). The screening trial is performed in accordance with the principles of the declaration of Helsinki. The demographic and clinical characteristics of participates in this study are shown in Table 1. Propensity score matching analysis was performed to adjust for differences in baseline characteristics with the covariate of age using the "MatchIt" R package.

**Table 1:** Demographic and clinical characteristics of participates in this study

Variable	Controls (*n* = 34)	PAD (*n* = 145)	*P*-value	PAD after propensity score matching (*n* = 34)	*P*#-value
Age, years	62.68 ± 9.69	69.93 ± 9.21	*P* < 0.001	65.47 ± 9.59	*P* = 0.092
Gender (male)	19 (55.9%)	116 (80.0%)	*P* = 0.003	30 (88.2%)	*P* = 0.006
BMI (kg/m2)	26.63 ± 5.04	27.68 ± 4.44	*P* = 0.025	28.26 ± 4.60	*P* = 0.038
Current/ex-smokers	11 (32.4%)	88 (60.7%)	*P* = 0.003	21 (61.8%)	*P* = 0.015
Rutherford Categories			NA		NA
Category 0	0	33 (22.8%)		9 (26.5%)	
Category 1	0	34 (23.4%)		9 (26.5%)	
Category 2	0	34 (23.4%)		7 (20.6%)	
Category 3	0	34 (23.4%)		7 (20.6%)	
Category 4	0	10 (6.9%)		2 (5.9%)	
**Baseline characteristics**					
Hypertension	14 (41.2%)	121 (83.4%)	*P* < 0.001	29 (85.3%)	*P* < 0.001
Coronary heart disease	9 (26.5%)	84 (57.9%)	*P* = 0.001	15 (44.1%)	*P* = 0.128
Renal insufficiency (eGFR < 60 mL/min/1.73m2)	2 (5.9%)	34 (23.4%)	*P* = 0.019	7 (20.6%)	*P* = 0.150
Heart failure	7 (20.6%)	68 (46.9%)	*P* = 0.005	10 (29.4%)	*P* = 0.401
Carotid artery stenosis > 30%	3 (8.8%)	55 (37.9%)	*P* = 0.001	7 (25%)	*P* = 0.163
Diabetes	3 (8.8%)	45 (31%)	*P* = 0.009	10 (29.4%)	*P* = 0.062
Atrial fibrillation	0	19 (13.1%)	*P* = 0.026	1 (2.9%)	*P* = 1
Creatinine, µmol/l	76.62 ± 14.27	96.63 ± 49.13	*P* = 0.001	90.12 ± 26.66	*P* = 0.018
eGFR, ml/min per 1.73m2	85.57 ± 14.92	73.13 ± 21.29	*P* = 0.003	78.78 ± 19.94	*P* = 0.117
NT-proBNP	109.19 ± 97.86	622.43 ± 2912.20	*P* < 0.001	253.47 ± 395.07	*P* = 0.092
hs Troponin	8.43 ± 5.68	15.18 ± 11.54	*P* < 0.001	12.09 ± 8.72	*P* = 0.011
total cholesterol, mmol/L	4.88 ± 1.24	4.11 ± 1.49	*P* < 0.001	4.08 ± 1.25	*P* = 0.010
Triglyceride, mmol/L	1.44 ± 0.94	1.88 ± 2.30	*P* = 0.077	1.96 ± 1.38	*P* = 0.050
LDL-Cholesterol, mmol/L	2.82 ± 0.92	2.19 ± 0.98	*P* < 0.001	2.08 ± 0.98	*P* = 0.002
HDL-Cholesterol, mmol/L	1.51 ± 0.45	1.28 ± 0.37	*P* = 0.005	1.29 ± 0.40	*P* = 0.031
Lipoprotein(a), mg/dl	25.29 ± 36.21	30.47 ± 38.28	*P* = 0.322	24.36 ± 34.62	*P* = 0.980
Ankle brachial index (ABI)	1.0 ± 0.14	0.86 ± 0.18	*P* < 0.001	0.81 ± 0.23	*P* < 0.001
Flow mediated dilation (FMD), %	9.6 ± 2.11	8.65 ± 2.68	*P* = 0.055	9.19 ± 2.27	*P* = 0.439
Heart rate, beat/min	66.71 ± 8.79	69.10 ± 11.41	*P* = 0.271	68.19 ± 11.36	*P* = 0.617
Systolic blood pressure, mmHg	132.5 ± 11.94	139.80 ± 19.66	*P* = 0.144	141.23 ± 18.77	*P* = 0.143
Diastolic blood pressure, mmHg	79.88 ± 9.06	78.15 ± 11.76	*P* = 0.119	79.42 ± 10.03	*P* = 0.404
**Treadmill testing**					
Pain-free walking distance, m	700 ± 0	367.07 ± 250.63	*P* < 0.001	452.48 ± 264.19	*P* < 0.001
Maximum walking distance, m	700 ± 0	440.76 ± 228.36	*P* < 0.001	532.13 ± 221.33	*P* < 0.001
Walking time < 6 min	0	53 (38.7%)	*P* < 0.001	7 (10.8%)	*P* = 0.004

*P*-value means compare Controls with PAD group; *P*#-value means compare Controls with PAD after propensity score matching group

### Isolation of peripheral blood mononuclear cells (PBMCs), RNA Extraction and quantitative real-time polymerase chain reaction (qRT-PCR)

The blood samples from each participant were processed to isolate PBMCs using Ficoll solution as described previously [16]. Briefly, 15 ml peripheral venous blood samples were collected in three vacutainer EDTA tubes. Next, 10 ml samples were diluted 1:1 with PBS, and carefully layered onto the Ficoll-Paque density gradient media (GE Healthcare) at a ratio of 4:3 and centrifuged at 400 g for 25 min. The middle layer containing PBMCs was collected and washed twice with PBS, and stored at -80 °C for RNA isolation. 5 ml samples were centrifuged at 1000 g for 10 min, the plasma was collected at -80 °C until use.

Total RNA was extracted from PBMCs by using the Trizol reagent (Thermo Fisher Scientific) according to the manufacturer’s instructions. The quality and quantity of total RNA were measured using NanoDrop-2000 spectrophotometer (NanoDrop Technologies). RNA samples with a 260/280 ratio between 1.8—2.0 were considered qualified. 1 μg of total RNA was reverse transcribed into cDNA using QuantiTect Reverse Transcription Kit (QIAGEN). The mRNA levels of the target gene were analyzed by real-time polymerase chain reaction using the LightCycler® 96 Real-Time PCR System (Roche). The relative expression of Mrna was calculated by 2^−ΔΔCt^ method. All the primers were synthesized and purchased from Eurofins Genomics Germany GmbH (available in Additional file 1: Table S1).

### Western blotting

PBMCs were lysed with RIPA lysis buffer and protein concentrations determined with the BCA Protein Assay kit according to the manufacturer’s protocol. Subsequently, 20 µg of protein/well were separated using 12% or 15% SDS‑PAGE. The proteins were subsequently transferred to nitrocellulose membranes at 250 mA for 15 to 60 min. Then membranes were blocked with Tris‑buffered saline containing 0.1% Tween 20 (TBST) and 5% non‑fat milk powder for 2 h at room temperature. After that, the membranes were incubated with primary antibodies overnight at 4˚C on a shaker set at a slow speed, then washed thrice with TBST and incubated with secondary antibody for 2 h at room temperature. After washing thrice, ECL substrate was added to the nitrocellulose membrane. The signal was detected using VWR Imaging Capture System. Band density was quantified with ImageJ software. All antibodies (P62, beclin1, LC3B, GAPDH, and HRP) were purchased from Abcam.

### Human chemokine antibody array

The Human Chemokine Antibody Array-Membrane from Abcam was used to detect 38 chemokines in plasma according to the manufacturer’s instructions. In brief, chemokines receptor-coated membranes were incubated with diluted plasma overnight at 4 °C. After appropriate washing and antibody incubating using reagents provided by the manufacturers, membranes were imaged on the VWR Imaging Capture System. The densitometric analysis of chemokine spots was performed using the MicroArray Profile plugin for ImageJ.

### Enzyme-linked immunosorbent assay (ELISA)

Plasma GRO and NAP2 levels were measured using the human GRO ELISA kit (RayBio) and human NAP2 ELISA kit (Abcam) according to the manufacturer’s instructions.

### Flow mediated dilation (FMD) measurement

FMD measurement is regarded as the gold standard method for evaluation of endothelial function. Here, we used the AngioDefender (Everist Health) medical device to measure participants’ FMD, which allows for a precise, standardized, and automated value by oscillation technique and has been proven to show less measurement errors in compared with classical ultrasound techniques measurement.

### Gardner treadmill testing

A treadmill and the Gardner treadmill protocol were used to assess patients’ walking capacity as previously reported [16]. Briefly, treadmill speed was maintained at 3.2 km/h with gradient increased 2 degrees every 2 min. This testing was performed under the supervision of a physician. The patient was secured with a drop stop device (safety bar with chest harness). Pain-free walking distance, maximum walking distance and walking time were recorded.

### The construction of nomogram

Reduced walking capacity is one of the main clinical features of PAD. Here, we construct a nomogram to predict the impaired walking capacity. Logistic regression model with least absolute shrinkage and selection operator (LASSO) selection method was used to identify relevant factors, and then, the nomogram was constructed using the “rms” R packages. The calibration plot was used to evaluate the accuracy of the nomogram, and receiver operating characteristic (ROC) curve and area under the ROC curve (AUC) value were used to evaluate the predictive efficacy.

### Statistical analysis

The statistical analyses and plots were performed using R software (version 4.1.0) and R Studio. For independent-samples, Student’s t test was used. Continuous variables were compared using the t test if data distribution met the criteria for normality; otherwise, the Wilcoxon rank sum test was used. P < 0.05 was considered statistically significant.

## Results

### Differentially expressed autophagy-related genes in PAD

**Fig. 1:**
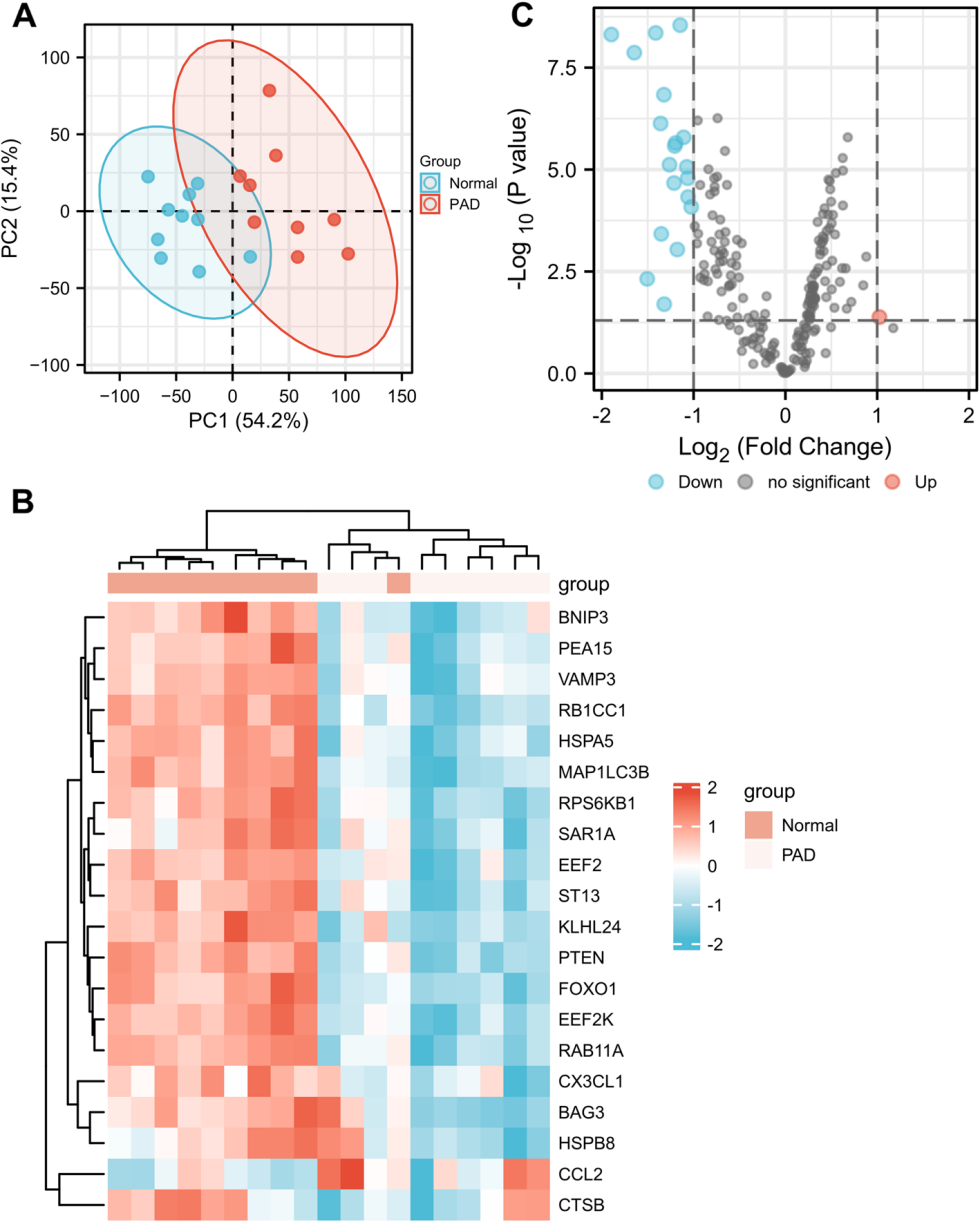
Differentially expressed autophagy-related genes in PAD and healthy samples. **A** Principal component analysis for GSE57691. **B** Heatmap of the 20 differentially expressed autophagy-related genes in PAD and healthy samples. **C** Volcano plot of the 20 differentially expressed autophagy-related genes. The red dots represent the significantly up-regulated genes and the blue dots indicate the significantly down-regulated genes

First, we evaluated the quality of the data, and performed a PCA. The data in GSE57691 exhibit satisfactory repeatability (Fig. 1A). In order to screen for differentially expressed autophagy-related genes, we next analyzed the expression of 232 autophagy-related genes in GSE57691, and 20 autophagy-related genes were identified using the criteria of adjusted *P*-value < 0.05 and |log2FC|≥ 1, including 1 up-regulated genes and 19 down-regulated genes, which were presented in heatmap and volcano plot (Fig. 1B and C).

### PPI network and correlation analysis of the differentially expressed autophagy-related genes

**Fig. 2:**
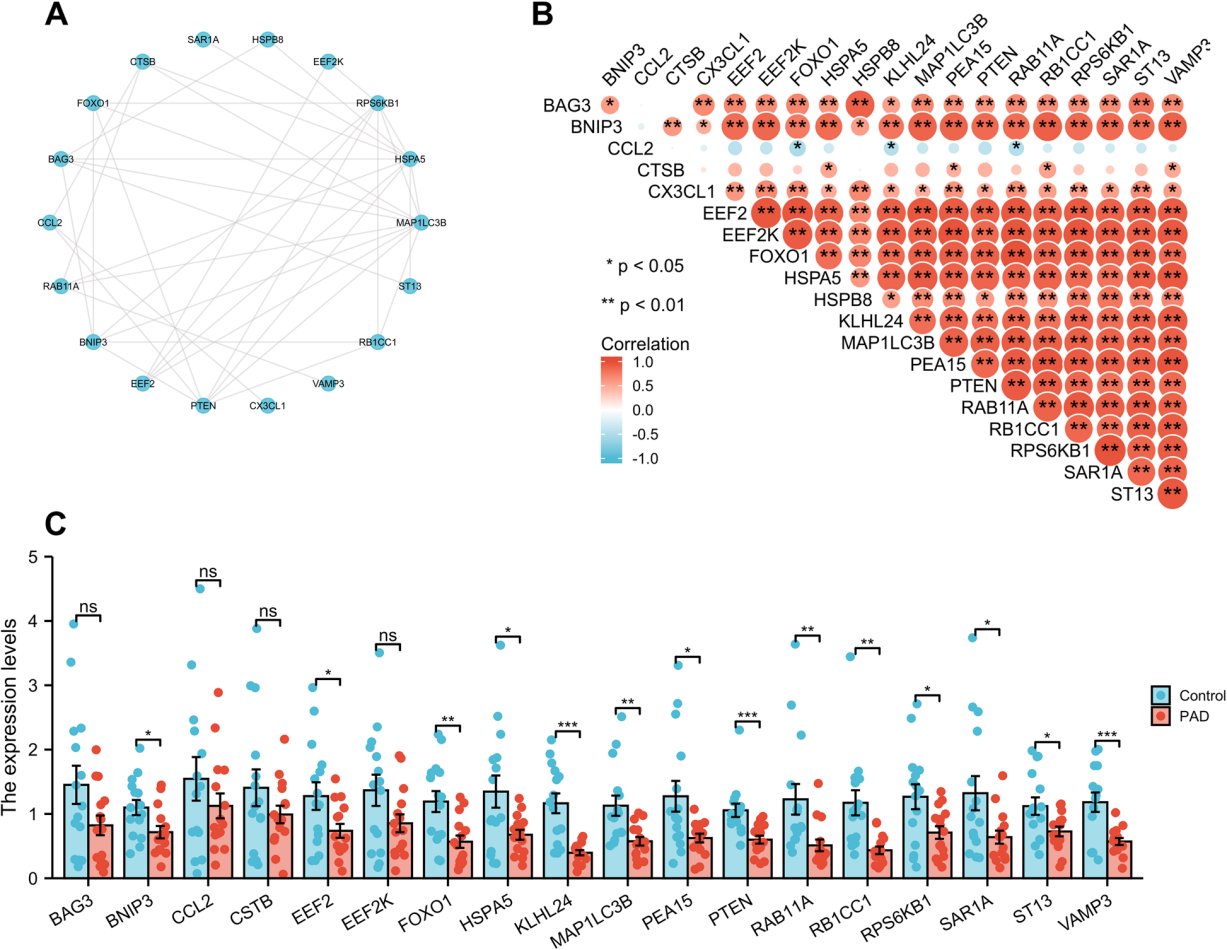
PPI, Spearman correlation analysis, and validation of the 20 differentially expressed autophagy-related genes. **A** The PPI among 20 differentially expressed autophagy-related genes. **B** Spearman correlation analysis of the 20 differentially expressed autophagy-related genes. **C** Histogram of mRNA expression levels of the autophagy-related genes in WalkByLab participates. Samples with Ct values of reference genes greater than 25 were removed. *P < 0.05; **P < 0.01; ***p < 0.001; ns, non-significant

To determine the interactions among differentially expressed autophagy-related genes, we performed PPI analysis. The proteins corresponding to the genes interact biochemically with each other in at least one and up to 10 different ways (Fig. 2A). To explore the expression correlation of these autophagy-related genes, Spearman correlation analysis was performed, yielding a potential interaction of 20 differentially expressed autophagy-related genes in the GSE57691 dataset (Fig. 2B).

### Validation of differentially expressed autophagy-related genes in WalkByLab registry PAD patients

The expression levels of these differentially expressed autophagy-related genes were further validated by qRT-PCR analyses of PBMCs from our cohort of WalkByLab Brandenburg (PAD, *n* = 18; non-PAD controls, *n* = 18. Clinical characteristics available in Additional file 1: Table S2). Expression levels of BNIP3, EEF2, FOXO1, HSPA5, KLHL24, MAP1LC3B, PEA15, PTEN, RAB11A, RB1CC1, RPS6KB1, SAR1A, ST13, and VAMP3 genes were significantly lower in PAD patients as compared to non-PAD controls (Fig. 2C). The BAG3, CCL2, CSTB, and EEF2K genes tended to be less expressed in the PAD group but did not reach statistical significance. However, Expression levels of CX3CL1 and HSPB8 genes were very low, so we did not include them in the statistical analysis.

### GO and KEGG enrichment analysis of the differentially expressed autophagy-related genes

**Fig. 3:**
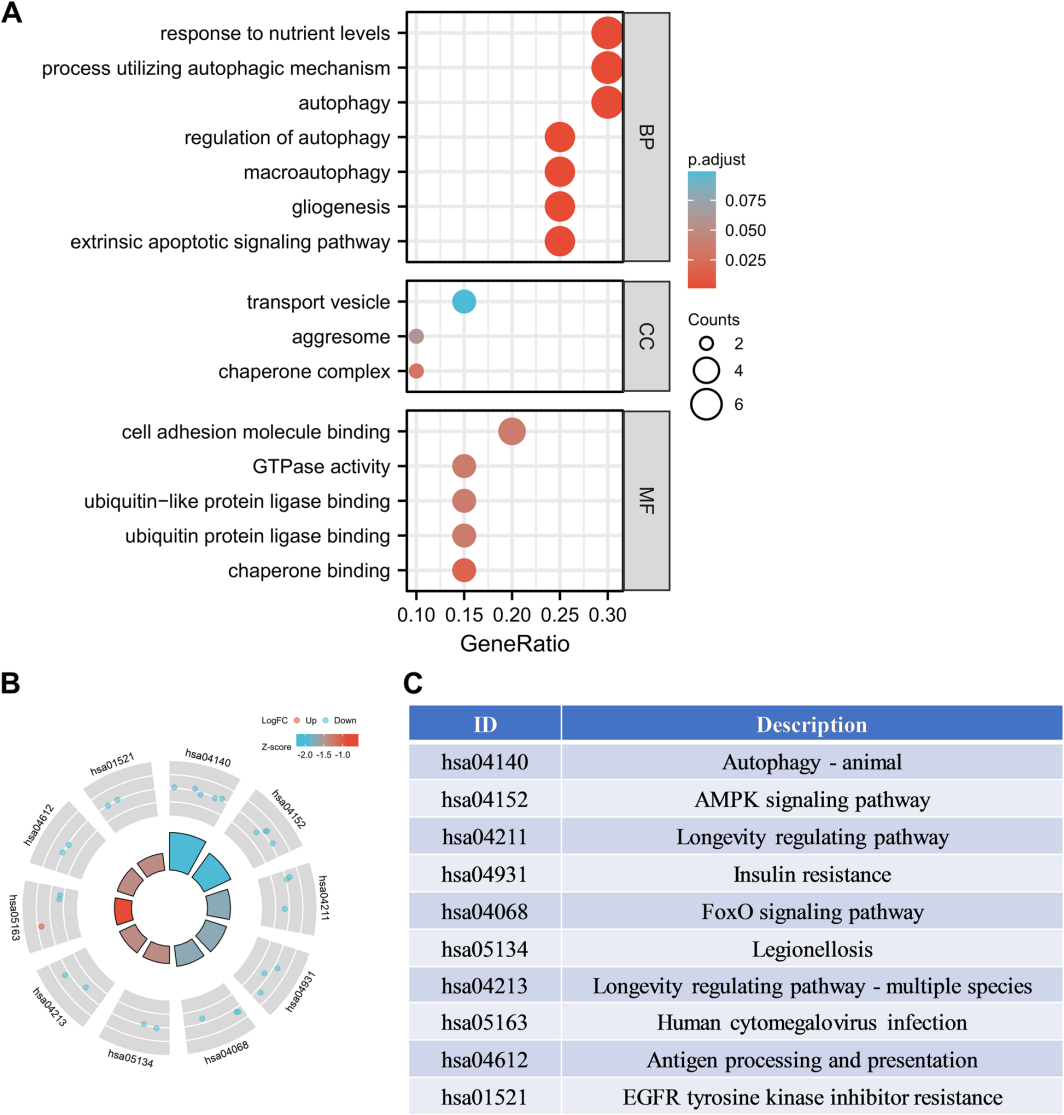
GO enrichment and KEGG analysis of 20 differentially expressed autophagy-related genes. **A** GO enrichment analysis. **B** and **C** KEGG analysis

To analyze the potential biological functions of these differentially expressed autophagy-related genes, we conducted GO and KEGG enrichment analysis. The results revealed that the most significant GO enriched terms are linked to the biological functions as follows: 1) response to nutrient levels, process utilizing autophagic mechanism, autophagy (biological process); 2) transport vesicle, aggresome, chaperone complex (cellular component); and 3) cell adhesion molecule binding, GTPase activity, ubiquitin-like protein ligase binding (molecular function) (Fig. 3A; Additional file 1: Table S3). In KEGG enrichment analysis, the differentially expressed autophagy-related genes are mainly involved in the process of autophagy and AMPK signaling pathway (Fig. 3B and C; Additional file 1: Table S4).

### Autophagy levels of PBMC in WalkByLab registry PAD patients

So far, our analysis revealed that autophagy-related genes’ mRNAs are expressed at low levels in PBMCs of PAD patients and their potential biological functions are mainly associated with autophagy according to GO and KEGG analysis. Next, we assessed the expression of important autophagy markers (p62, beclin1, and LC3B) to explore the autophagy level between PAD and health.

**Fig. 4:**
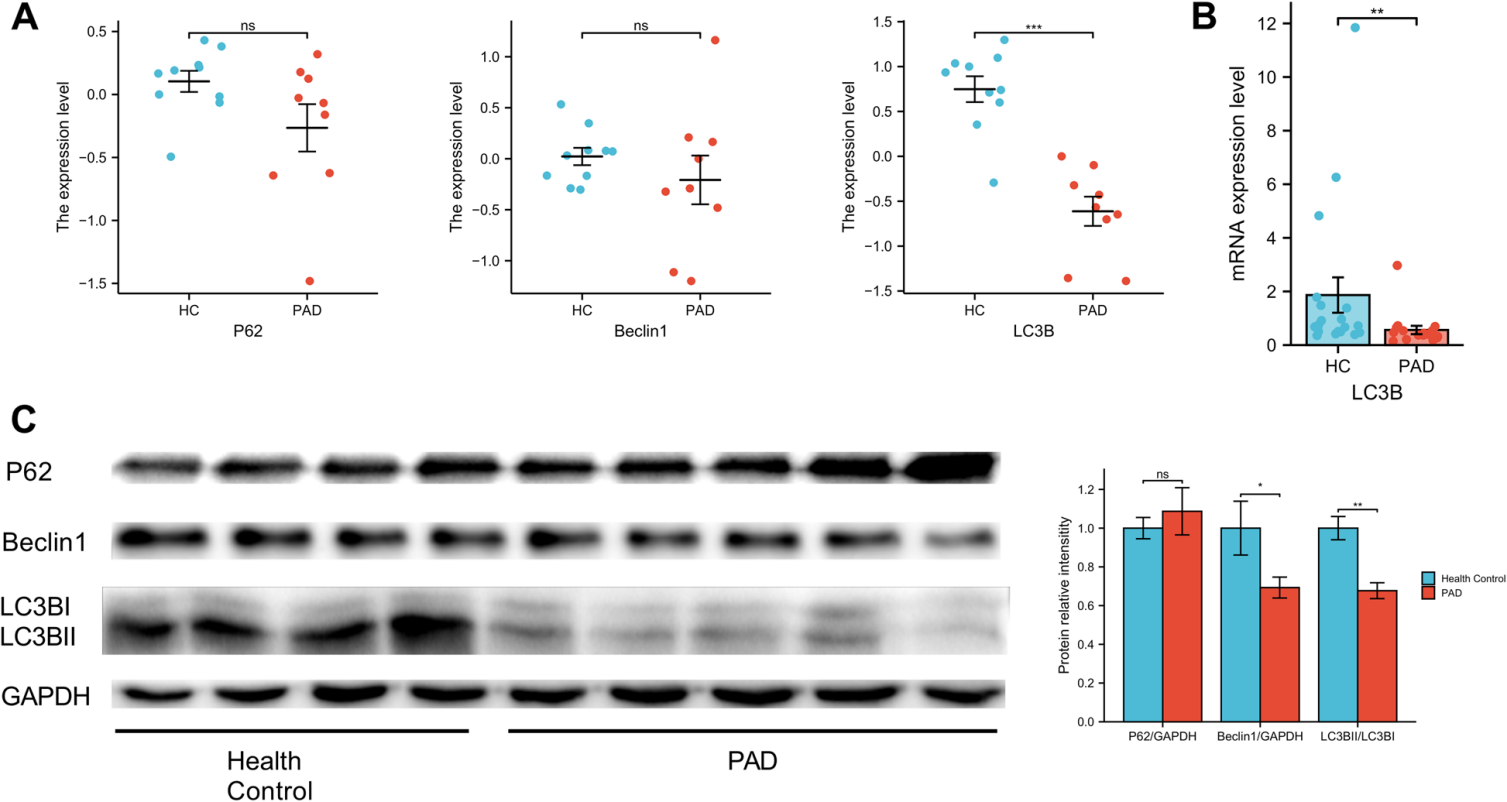
Autophagy levels in PAD patients and healthy controls (HC). **A** mRNA levels of autophagy markers (p62, Beclin1, and LC3B) in GSE57691. **B** qRT-PCR analysis of mRNA levels of autophagy marker LC3B in WalkByLab participates. **C** Western blotting analysis of protein levels of autophagy markers (p62, Beclin1, and LC3B) in WalkByLab participates. *p < 0.05, **p < 0.01, ***p < 0.001, ns, non-significant

First, the mRNA levels of p62, beclin1, and LC3B were examined in GSE57691 No significant differences in the mRNA levels of p62 and beclin1 were detected between PAD patients and healthy controls. However, the gene expression level of LC3B was found to be significantly decreased in PAD patients (Fig. 4A). Next, we validated a possible deregulation of LC3B mRNA expression in PBMCs from WalkByLab patients using qRT-PCR. LC3B in WalkByLab participates was less expressed in the PBMCs of patients with PAD (Fig. 4B). Finally, we assessed the protein levels of p62, beclin1 and LC3B in the PBMCs of WalkByLab participates. It turns out that there was a significant decrease in beclin1 and LC3B-II protein levels in PAD patients as compared to healthy controls (Fig. 4C). There was a trend of increased p62 level, but it was not statistically significant. In summary, results showed that protein levels of autophagy markers were decreased in PBMCs from patients with PAD.

### Quantitative analysis of immune microenvironment

**Fig. 5:**
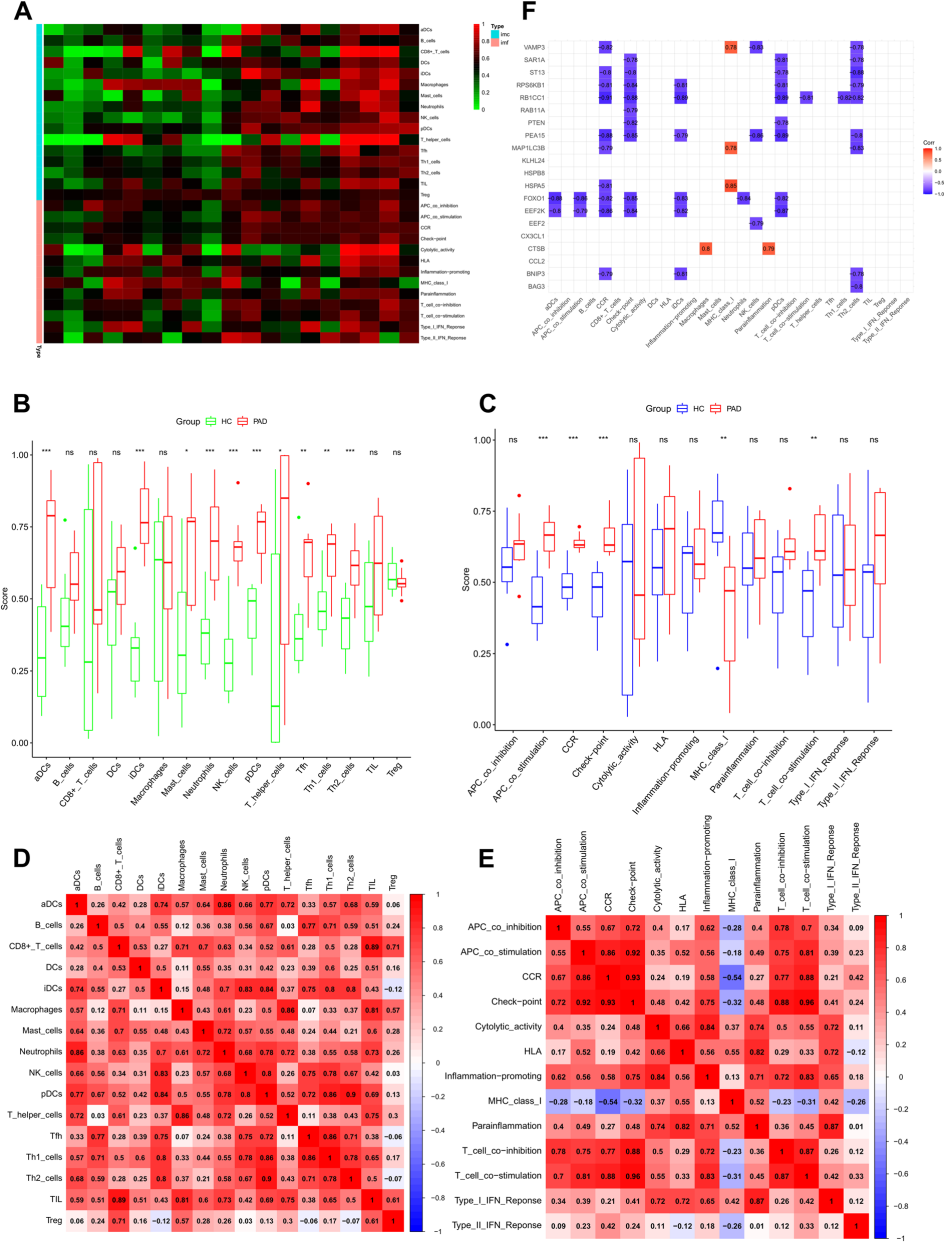
The landscape of immune microenvironment between PAD patients and healthy controls based on ssGSEA. **A** A heatmap of 16 immune infiltration cells and 13 immune-related pathways. **B** Boxplot of comparisons of 16 immune infiltration cell enrichment scores. **C** Boxplot of comparisons of 13 immune-related pathways enrichment scores. **D** Correlation matrix of ssGSEA scores of immune cells. **E** Correlation matrix of ssGSEA scores of immune-related pathways. **F** Correlation analysis between the expression of 20 differentially expressed autophagy-related genes and immune microenvironment. Red means positive correlation and blue means negative correlation. *p < 0.05, **p < 0.01, ***p < 0.001, ns, non-significant

PAD is a systemic disease of the arteries, which manifests in the periphery (e.g. legs, feet) but usually affects the majority of all body arteries. PAD is also an inflammatory disease, and the degree of systemic inflammation is even greater than in CAD due to the large number of arteries affected [17]. Thus, we performed a ssGSEA here to quantify the immune microenvironment of the artery wall and compared tissue from PAD patients with healthy controls. ssGSEA revealed 16 types of immune cells and 13 types of immune-related pathways in each sample, which is shown in the heat map (Fig. 5A). A higher level of immune cell infiltration (including aDCs, iDCs, Mast cells, neutrophils, NK cells, pDCs, T helper cells, Tfh, Th1, and Th2 cells) was identified in PAD as compared with healthy controls (Fig. 5B). Moreover, more active immune-related pathways (including APC co-stimulation, cytokine-cytokine receptor (CCR) interaction, check point, MHC class I, and T cell co-stimulation activity) were observed in PAD as compared with healthy controls (Fig. 5C). Further Spearman’s correlation analysis showed that the identified subset of infiltrating immune cell subgroups and active immune-related pathways were moderately to strongly correlated, respectively (Fig. 5D and E).

Next, we analyzed whether these 20 differentially expressed autophagy-related genes were correlated with immune function in PAD. We found that most autophagy-related genes (except for KLHL24, HSPB8, CX3CL1 and CCL2) correlated with immune cells infiltration and immune-related pathways (Fig. 5F). Among them, RB1CC1 and FOXO1 genes showed the highest reported number of relationships with immune microenvironment. Most infiltrating immune cells linked to autophagy-related genes were PBMCs such as iDCs (involved genes *n* = 6), pDCs (*n* = 8) and Th2 cells (*n* = 9). The top immune-related pathways associated with autophagy-related genes were found to be CCR interactions (*n* = 10), as demonstrated in Fig. 5F. Specifically, the following genes were identified as being involved in these pathways: VAMP3, ST13, RPS6KB1, RB1CC1, PEA15, MAP1LC3B, HSPA5, FOXO1, EEF2K, and BNIP3.

### Expression level of chemokines in plasma

Since CCR interaction was the top active immune-related pathway that linked to autophagy-related genes in the ssGSEA analysis, and combined with the results that autophagy-related genes and autophagy level were decreased in PAD patients’ PBMC, we suspect that CCR interaction might play an important role in the development and progression of PAD. Among CCR interactions, chemokines are best known for their ability to stimulate the migration of leukocytes. Since leukocytes are central to the progression of PAD and to our analysis of autophagy in PBMCs, chemokines play a particularly central role in our investigation.

**Fig. 6:**
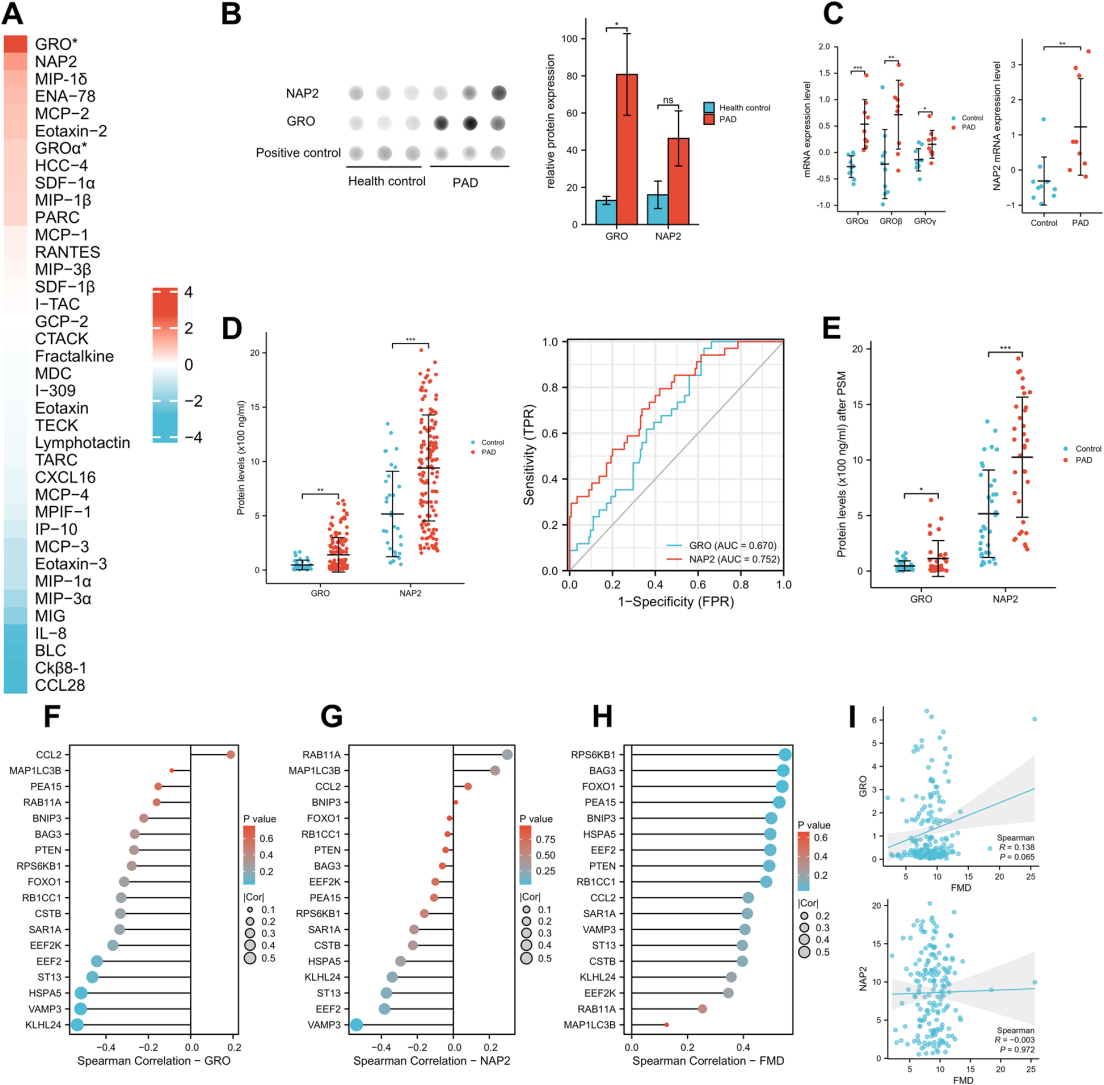
The plasma levels of chemokines in PAD and control groups and the relationship between autophagy-related genes, chemokines and endothelial function. **A** The expression profiles of human chemokines in PAD and health controls’ plasma in WalkByLab participates. Chemokines are ranked according to the value of the log2-fold changes between the two groups. **B** The relative protein expression of GRO and NAP2 in PAD and health controls. **C** The mRNA expression levels of the three subtypes of GRO and NAP2 in GSE57691. **D** The plasma levels of GRO and NAP2 as well as their diagnostic values for PAD in the whole cohort of 179 participants. **E** The plasma levels of GRO and NAP2 in the PSM cohort of 68 participants. **F**–**H** Spearman correlation analysis between autophagy-related genes and chemokines and FMD. **I** Spearman correlation analysis between chemokines and FMD. *P < 0.05; **P < 0.01; ***p < 0.001; ns, non-significant

Hence, we used the human chemokine antibody array to explore the plasma levels of chemokines (3 PAD and 3 health controls) and found that growth-related oncogene (GRO) and neutrophil activating protein2 (NAP2) chemokines had highest fold change expression between the two groups (Fig. 6A and B). The mRNA levels of GRO family (GROα, GROβ and GROγ) and NAP2 were also highly expressed in PAD patients in GSE57691 (Fig. 6C). Thus, we next utilized ELISA kits to measure the plasma expression levels of GRO and NAP2 in WalkByLab registry cohort of 179 participants. GRO and NAP2 levels were significantly higher in PAD patients’ plasma, and NAP2 (AUC: 0.752) demonstrated a better diagnostic performance for PAD patients as compared with GRO (AUC: 0.670) (Fig. 6D). Similar results were also shown in the propensity score matching analysis cohort after excluding the potential influence of age (Fig. 6E).

### The relationship between autophagy-related genes, chemokines and endothelial function

Spearman correlation analysis, which investigate the potential pairwise correlation between these three factors, showed that autophagy-related genes KLHL24, VAMP3, HSPA5, and ST13 were moderately negative correlated with chemokine GRO (|Cor|> 0.4) (Fig. 6F), and showed mild to moderate negative correlation with chemokine NAP2 (|Cor|> 0.3) (Fig. 6G). Fourteen autophagy-related genes were moderately positive correlated with FMD (Cor > 0.4) (Fig. 6H). However, the chemokines GRO and NAP2 were not correlated with FMD (*P* > 0.05) (Fig. 6I).

### The correlation between plasma chemokines and walking capacity

**Fig. 7:**
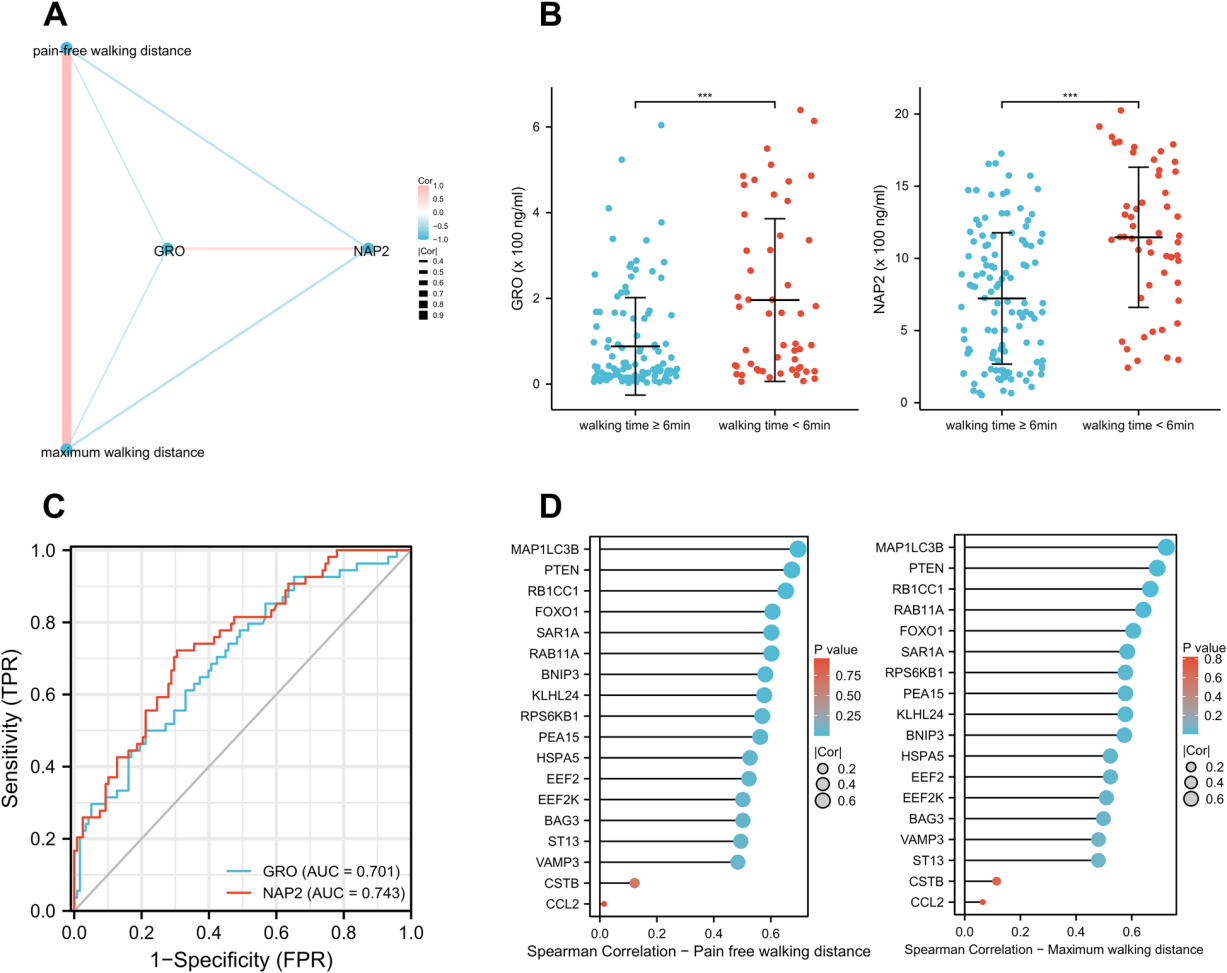
The relationship between chemokines, autophagy-related genes, and treadmill walking capacity. **A** The correlation network plots for pairwise correlations among GRO, NAP2 and walking distance in Gardner treadmill testing. **B** Participates with walking time less than 6 min in Gardner treadmill testing had higher levels of GRO and NAP2 protein. **C** The predict values of GRO and NAP2 for impaired walking capacity in Gardner treadmill testing. **D** Spearman correlation analysis between autophagy-related genes and walking distance in Gardner treadmill testing. *P < 0.05; **P < 0.01; ***p < 0.001; ns, non-significant

Gardner treadmill testing is a gold standard exercise stress test, providing clinically important diagnostic and prognostic information for patients with symptomatic PAD [18]. The results of the Spearman correlation analysis revealed that there were significant correlations between the levels of GRO and NAP2 and the walking capacity. Specifically, the GRO level showed a mild negative correlation with pain-free walking distance (Correlation coefficient, Cor = -0.331) and maximum walking distance (Cor = -0.340). The NAP2 level demonstrated a mild to moderate negative correlation with pain-free walking distance (Cor = -0.387) and maximum walking distance (Cor = -0.403). Besides, there was a moderate positive correlation between the levels of GRO and NAP2 (Cor = 0.434), and a strong positive correlation between pain-free walking distance and maximum walking distance (Cor = 0.913). The correlation network plots for pairwise correlations among the studied variables are showed in Fig. 7A (all *P* < 0.05).

Furthermore, participates which showed during the walking test a walking time less than 6 min had higher levels of GRO and NAP2 protein (Fig. 7B). ROC analysis to determine the diagnostic performance in walking time less than 6 min showed that NAP2 (AUC: 0.743) had a higher efficacy than GRO (AUC:0.701) (Fig. 7C).

### The correlation between autophagy-related genes and walking capacity

Spearman correlation analysis showed that, except for CCL2 and CSTB genes, all other autophagy-related genes exhibited a significant positive correlation with walking distance, with correlation coefficients ranging from moderate to strong (0.40 < Cor < 0.73) (Fig. 7D). Specifically, MAP1LC3B gene demonstrated strong positive correlations with pain-free (Cor = 0.70) and maximum walking distance (Cor = 0.73).

### The correlation between plasma chemokines and major adverse cardiovascular events (MACE)

We reviewed the history of MACE occurrence, including stroke, transient ischemic attack, and myocardial infarction, among the enrolled participants. Our results showed that there was no statistical difference in NAP2, GRO, and ankle-brachial index (ABI) levels between the groups with and without the history of MACE occurrence (*P* > 0.5) (Additional file 2: Figure S1). The AUC of GRO was 0.529 and that of NAP2 was 0.516, which is marginally higher than that of ABI (AUC: 0.508). Overall, these biomarkers almost had little predictive power for MACE.

### Construction of a nomogram to predict impaired exercise tolerance

**Fig. 8:**
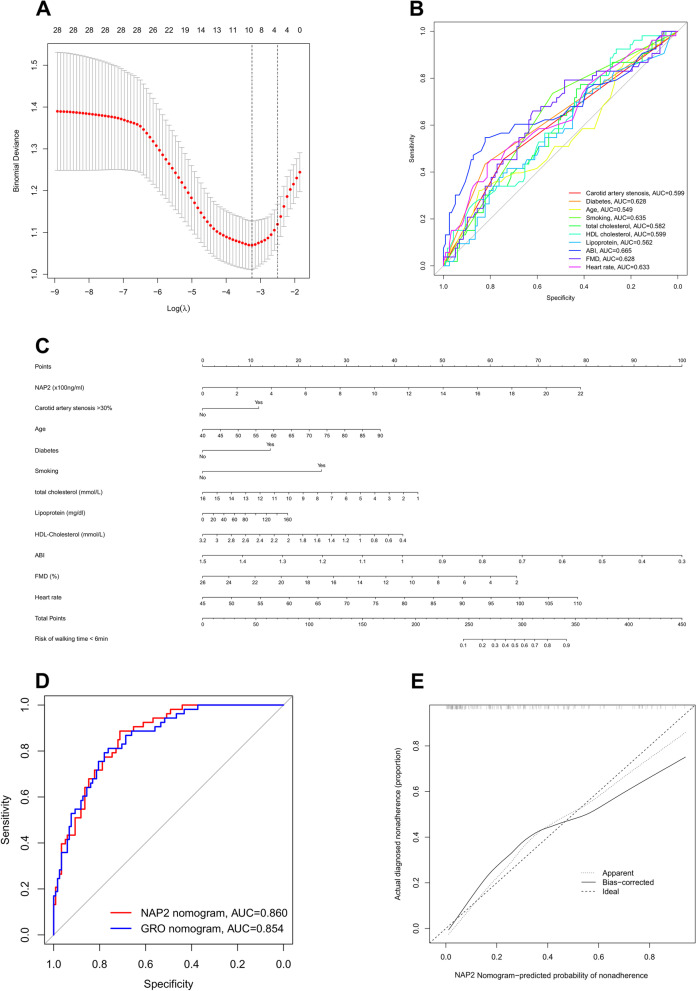
Construction of nomogram model. **A** LASSO regression analysis selected the potential variables. **B** ROC curve shows the predicted ability of the chosen variables. **C** Nomogram integrated with NAP2 predicting walking time < 6 min in Gardner treadmill testing. **D** ROC curve analysis compares the predicted efficacy of different nomogram models. **E** Calibration curves for nomogram predicted walking time < 6 min in Gardner treadmill testing

Nomogram is a powerful tool used for predicting the incidence of diseases or patients’ prognosis. Here, we constructed a nomogram model to predict impaired walking capacity. A walking time < 6 min in Gardner treadmill testing was defined as the endpoint. LASSO regression was performed firstly to select the most effective variables from all clinical features and ten clinical parameters were obtained (Fig. 8A). The respective AUC values of these parameters were shown in Fig. 8B.

Given the significant correlation between the levels of GRO and NAP2 and walking capacity, we selected these two factors as candidate variables for multivariate logistic regression analysis and nomogram construction (Fig. 8C and Additional file 2: Figure S2). Due to the limited sample size available for the autophagy-related genes in our study, we opted not to include them as candidate variables for analysis. ROC curves analysis showed that the nomogram integrated with NAP2 (AUC: 0.860) had a better predicted efficacy than nomogram with GRO (AUC: 0.854) (Fig. 8D). Finally, calibration plots were used to visualize the performances of the nomograms. The calibration plot confirmed the performance of our predictive model (Fig. 8E).

## Discussion

Autophagy is a cellular self-recycling process, which is involved in both vascular homeostasis as well as physiological and pathological arterial remodeling. Physiological autophagy serves as a protective mechanism to maintain normal cardiovascular function, whereas disordered autophagy contributes to the development and progression of diseases [19]. It seems to play a protective role in the early phase of atherosclerosis, as deletion of autophagy genes (Atg5) accelerates the progression of atherosclerosis in murine models [20]. In clinical studies, protein levels of autophagy‑associated protein beclin1 and light chain 3 (LC3) were decreased in PBMCs from patients with acute myocardial infarction compared with healthy controls or patients suffering from stable angina pectoris [21]. Based on these findings, it is speculated that acute cardiovascular events and increased severity of disease might lead to a reduction in autophagy and vice versa [6, 21]. However, the role of autophagy in cardiovascular disease development is not entirely clear, in particular, there are little study related to PAD.

In this study, we identified 20 potential autophagy-related genes in PAD through bioinformatic analysis, and validated changes in the expression of these genes in peripheral blood samples from the WalkByLab registry trial. All identified genes were expressed at low levels or had the tendency of decreasing in PAD patients’ PBMCs, which indicated a role of autophagy-related genes in PAD. The GO and KEGG enrichment analysis further revealed that these autophagy-related genes might be involved in autophagy activity and control of autophagy activation. Indeed, western blot analysis demonstrated that autophagic marker proteins beclin1 and LC3B levels were significantly decreased in the PBMCs of PAD patients. Beclin1 and LC3B are common biomarkers of autophagy, which are widely used to evaluate the level of autophagy. Our study now provided evidence that PAD is linked to a reduced expression level of these autophagy-related genes and a decreased autophagy marker protein level.

Inflammation is one of the main features of PAD. Autophagy plays an important role in inflammation by regulating the development, homeostasis, and survival of inflammatory cells such as macrophages, neutrophils, and lymphocytes. Autophagy also influences the transcription, processing, and release of a variety of cytokines and is regulated by cytokines [22]. Autophagy can trigger a dynamic response to inflammation. Active autophagy suppresses excessive inflammatory response by inhibiting inflammasome activation, facilitating damage-associated molecular patterns and damaged mitochondria clearance, and destroying inflammatory mediators [23]. Suppressed autophagy resulted in an enhanced inflammatory response [24].

Thus, we explored the overall characteristics of the immune microenvironment of artery tissue from PAD patients and healthy populations and the association with autophagy-related genes. We found higher level of immune cell infiltration and more active immune-related pathways in PAD and that the CCR interaction was the most active immune-related pathway negatively associated to autophagy-related genes. This seemed to imply that the decreased autophagy will increase inflammation in PAD. Here, the cytokines include the chemokines, the PDGF family, and the TGF-β family [25]. Among CCR interactions, chemokines are best known for their ability to stimulate the migration of leukocytes (e.g. PBMCs) and chemokines in particular are known to play an important role in the pathophysiology of cardiovascular disease [26]. Chemokines bind to specific G protein-coupled receptors and exert distinct functions, such as regulating the activation of leukocytes and coordinating their trafficking to the sites of inflammation [27]. For example, chemokine RANTES level is a useful marker of CAD severity because elevated chemokine RANTES level in patients with stable angina may predict the high risk of plaque formation in early stages of atherosclerosis [28]. Therefore, we measured the expression profiles of human chemokines in plasma from PAD patients and healthy individuals to identify a potential role of chemokines in PAD pathophysiology. ELISA analysis of the WalkByLab participants showed that human chemokine GRO and NAP2 was highly expressed in PAD which was also demonstrated by a strict propensity score-matched analysis after adjustment for covariate of age. In addition, our analysis indicated that GRO and NAP2 may be promising diagnostic biomarkers for PAD, as reflected by their respective AUC values of 0.670 and 0.752. Moreover, our analysis showed that the levels of GRO and NAP2 were not associated with MACE (acute events), therefore indicating their specificity for a systemic PAD-related pathophysiology. Interestingly, our study also revealed a negative correlation between GRO and NAP2 levels and the expression of autophagy-related genes KLHL24, VAMP3, HSPA5, and ST13, suggesting a potential link between the dysregulation of autophagy and chemokine-mediated inflammation in PAD. Further investigation is needed to elucidate the mechanistic relationship between autophagy and GRO/NAP2 signaling in the context of PAD pathophysiology.

We next examined other crucial characteristics of PAD pathophysiology. Important cofactors and clinical parameters for the evaluation of PAD are endothelial function measured by FMD and walking distance measured by a Gardner treadmill test. Our study demonstrated that plasma GRO and NAP2 levels were not correlated with participants’ FMD, but were negatively correlated with the pain-free walking and maximum walking distance in Gardner treadmill testing. Besides, GRO and NAP2 were able to predict poor walking capacity (walking time < 6 min in Gardner treadmill testing) with respective AUC values of 0.701 and 0.743. Although FMD is a very valuable clinical parameter, it is also very sensitive to a variety of external influences, such as biological age, diet, or exercise level of the patient [29]. In clinical practice FMD is therefore not used for the primary diagnosis of PAD. However, the Gardner treadmill walk test is considered the gold standard to accurately determine the stage of PAD according to the Fontaine stage. To our knowledge, a role of GRO and NAP2 for PAD in the context of walking distance and walking capacity has never been demonstrated before. Previous studies have mainly focused on the role of GRO and NAP2 in cancer development and metastasis of various malignancies [30], whereas few reports have focused on cardiovascular disease, and even fewer in the context of PAD. In the study of Ku et al. [31], upregulated NAP2 was significantly associated with severity of coronary artery stenosis in patients with diabetes. Recently Wang et al. showed that elevated plasma NAP2 level was independently associated with critical limb ischemia, not with diabetes [32], however a connection with the walking capacity was not considered. Until now the deregulation of GRO was never shown before in the context of PAD. Chemokines GROα, GROβ, and GROγ constitute the three members of the GRO family [33], which together with NAP2, share the chemokine receptor CXCR2 as a common binding receptor [34]. GROγ was reported to be upregulated in acute coronary syndromes and related to the severity of the inflammatory response [35]. Moreover, a recent study demonstrated that CXCL3 (the coding gene of GROγ protein) gene was highly expressed in acute ischemic stroke and it can be recognized as a predictor for more infarct volume [36]. In summary, chemokines GRO and NAP2 appear to be useful biomarkers in PAD, particularly in predicting impaired walking ability.

Finally, we further analyzed and processed plasma GRO or NAP2 levels with the relevant clinical parameters obtained by LASSO analysis to build nomogram models for the prediction of poor walking capacity (walking time < 6 min in Gardner treadmill testing). The NAP2-derived nomogram showed a well predictive performance, with an AUC of 0.860, while the GRO-derived nomogram had a slightly lower AUC of 0.854. These evidence demonstrated that plasma NAP2 indeed could be used as a predictive biomarker and the derived nomogram model simply and accurately predicted impaired walking capacity in patients with PAD.

The here presented study has certain limitations that need to be acknowledged. Firstly, the sample size was relatively small, and therefore, it is important to exercise caution when generalizing the results. Secondly, this study was retrospective, and as such, there is a possibility of selection bias. Nevertheless, the retrospective nature of the study was necessary to pave the way for future prospective studies. Thirdly, a correlation between autophagy-related genes and chemokines with walking ability was discovered, suggesting that autophagy may play a role in the progression of PAD by regulating chemokines and inflammation. However, this potential mechanism requires further validation in animal models, and thus, future studies are necessary to shed more light on this issue.

## Conclusions

In conclusion, our study shed light on the interrelationships of autophagy and autophagy-related genes in the development of PAD, whcih were previously unknown. Our comprehensive analysis identified a set of 20 autophagy-related genes that were expressed at lower levels in PAD and demonstrated that the autophagy levels of PBMCs were significantly decreased in patients with PAD. This finding has significant implications for future clinical strategies and for deepening the understanding of disease onset and progression, as well as providing novel biomarkers. These results will serve to guide the clinic in developing new prognostic strategies for the evaluation of PAD. Our bioinformatics analysis further revealed a link between vascular inflammation and the effect of cytokines, particularly chemokines, with autophagy-related genes. This was also further validated in subsequent experiments. We found that GRO and NAP2 levels were elevated in the plasma of PAD patients, with a mild-to-moderate negative correlation between them and the autophagy-related genes KLHL24, VAMP3, HSPA5, and ST13. The effects of GRO and NAP2 on walking capacity in PAD have never been proven before. Our study reveals a mild-to-moderate negative correlation between chemokines and pain-free walking distance and maximal walking distance in the Gardner treadmill test. This suggests that reduced autophagy may influence the onset and progression of PAD (especially, the walking capacity) by affecting chemokines (inflammation). Finally, we constructed nomogram models that can predict poor walking capacity. The results showed that NAP2 is a promising biomarker for PAD patients, and its derived nomogram had stronger predictive potential than the GRO derived nomogram. This nomogram provides a valuable tool for clinicians to improve their understanding of a patient's disease risk and prognosis, thereby facilitating informed clinical decision-making. In all, our study provides new insights into the molecular mechanisms underlying PAD and may have implications for the development of new diagnostic and therapeutic approaches for PAD.

## Supplementary Information

**Additional file 1:** **Table S1.** Primer sequences. **Table S2.** Clinical characteristics of PAD patients and controls in the qRT-PCR analysis. **Table S3.** GO enrichment analysis of 20 differentially expressed autophagy-related genes. **Table S4.** KEGG pathway enrichment analysis of 20 differentially expressed autophagy-related genes.

**Additional file 2:** ** Figure S1. **The correlation between plasma GRO/NAP2 chemokines and major adverse cardiovascular events. **Figure S2.** Nomogram integrated with GRO predicting walking time<6min in Gardner treadmill testing.

**Additional file 3:** **Figure S1.** The original blot image of P62. **Figure S2.** The original blot image of Beclin1. **Figure S3.** The original blot image of LC3B. **Figure S4.** The original blot image of GAPDH.

## Data Availability

Publicly available datasets were analyzed in this study. This data can be found here: GEO database, accession number: GSE57691.

## Abbreviations

ABI: Ankle brachial index
AUC: Area under the ROC curve
BP: Biological process
CAD: Coronary artery disease
CC: Cellular component
CCR: Cytokine and cytokine receptors
ELISA: Enzyme linked immunosorbent assay
FC: Fold change
FMD: Flow-mediated dilation
GEO: Gene expression omnibus
GO: Gene Ontology
GRO: Growth-related oncogene
KEGG: Kyoto encyclopedia of genes and genomes
LASSO: Least absolute shrinkage and selection operator
LC3: Light chain
MACE: Major adverse cardiovascular events
MF: Molecular function
NAP2: Neutrophil activating protein2
PAD: Peripheral arterial disease
PBMCs: Peripheral blood mononuclear cells
PCA: Principal component analysis
PPI: Protein–protein interaction
qRT-PCR: Quantitative real-time polymerase chain reaction
ROC: Receiver operating characteristic
ssGSEA: Single sample gene set enrichment analysis
TBST: Tris‑buffered saline containing 0.1% Tween 20
