# Supplementary material for: Autophagy-related genes analysis reveals potential biomarkers for prediction of the impaired walking capacity of peripheral arterial disease
Source: BMC Med. 2023 May 18;21:186. doi: 10.1186/s12916-023-02889-5 (PMC10193747; doi:10.1186/s12916-023-02889-5)

Additional File 3: Figure S1 P62

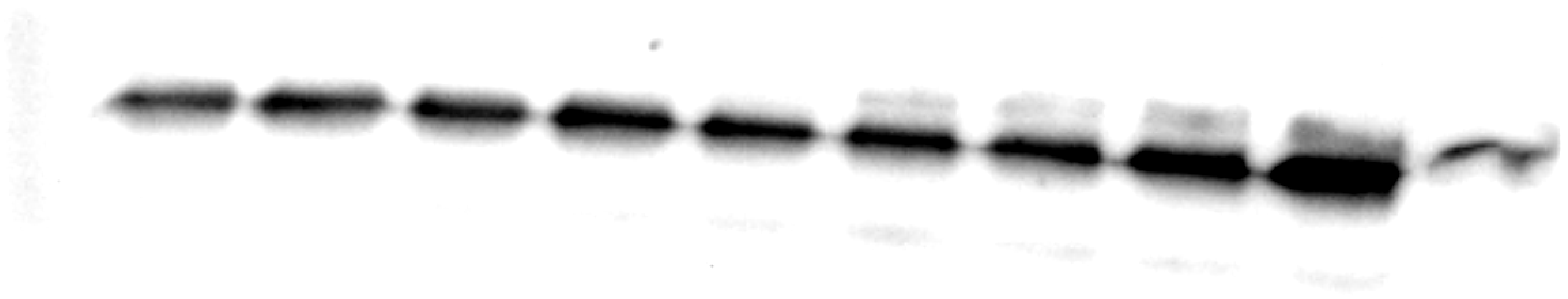

Additional File 3: Figure S2 Beclin1

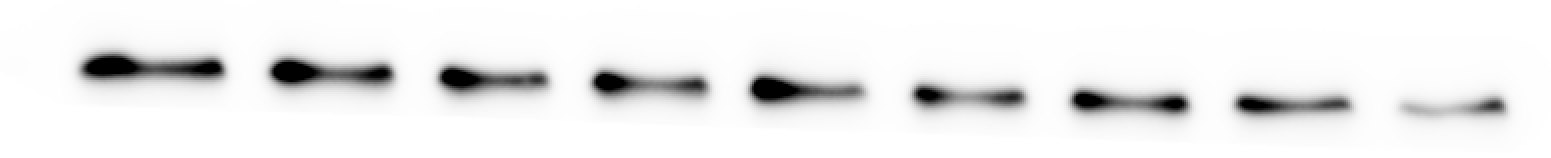

Additional File 3: Figure S3 LC3B

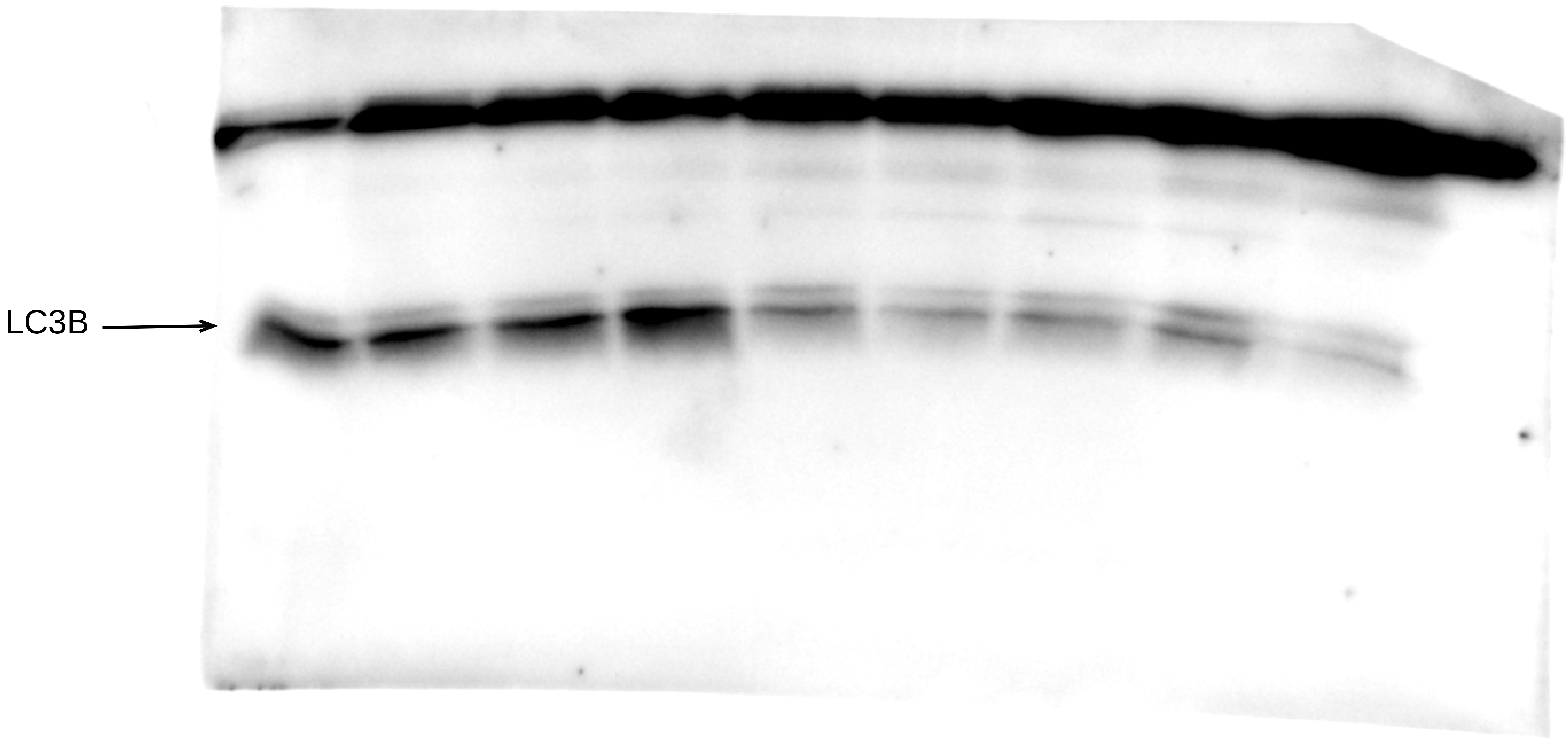

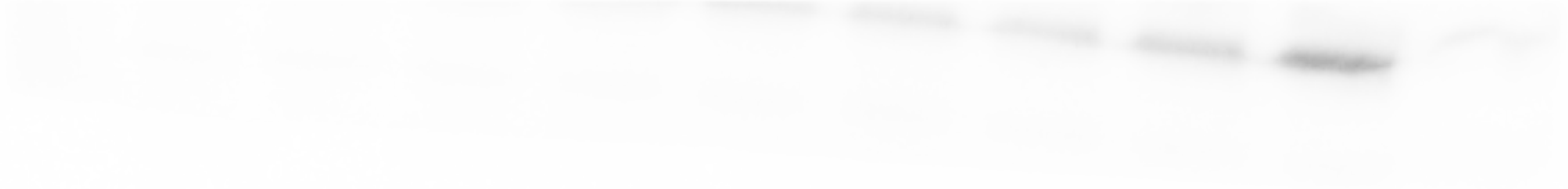

Additional File 3: Figure S4 GAPDH

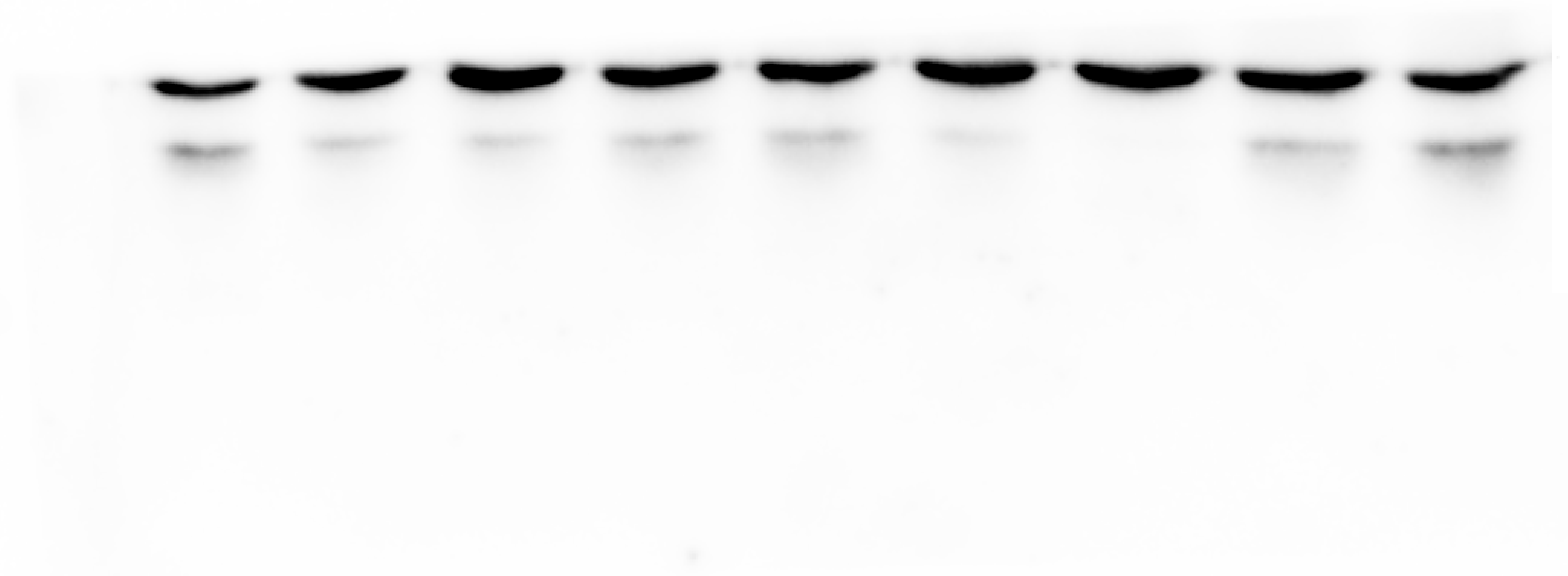

Supplement: Supplementary file 3 — Additional file 3: Figure S1. The original blot image of P62. Figure S2. The original blot image of Beclin1. Figure S3. The original blot image of LC3B. Figure S4. The original blot image of GAPDH. [file 12916_2023_2889_MOESM3_ESM.pdf]
